# Next Generation Rice Disease Research

**DOI:** 10.1186/s12284-021-00523-7

**Published:** 2021-09-27

**Authors:** Jonathan M. Jacobs, Guo-Liang Wang

**Affiliations:** 1 Department of Plant Pathology, The Ohio State University, Columbus, OH 43210 USA; 2 Infectious Disease Institute, The Ohio State University, Columbus, OH 43210 USA

Rice feeds over half the world’s population. In the face of climate change and global population increases, this essential food crop will encounter new challenges with environmental, pest and pathogen pressures. This special issue targets understanding biotic stress and developing novel control technologies in rice in a changing future.

Many pathogens cause important diseases in rice, leading severe yield losses worldwide (Liu et al. 2014). Disease management must factor in the phytobiome or all the biological entities promoting or inhibiting disease (Leach et al. 2017). The Next Generation Rice Disease Research Special Issue highlights features work from Roman-Reyna and colleagues (2020) at the International Rice Research Institute that describes the rice microbiome from the wealth of data from the 3000 Rice Genomes Project (The 3 2014). This work advances our perspectives on how we can consider associations between rice genotypes and microbiomes at a scale never explored. Future microbiome research can build off this work as a model for large-scale analysis linking rice traits to the microbes that inhabit the rice environment.

Translational biology to support rice farmers and disease management depends on a strong basic knowledge of host-microbe interactions. The Next Generation Rice Disease Research Special Issue includes three reviews highlighting important insights and advances in basic rice biotic stress research. Specific pathosystems such as sheath blight limit rice production, and Li et al. (2021) describe ways to use plant–microbe interactions research to gain insights into disease management. Vo et al. (2021) showcase the advances in proteomics and metabolomics. They describe not only how both have advanced our basic understanding of rice biotic stress but also provide specific metabolic targets that could help with disease management. A review by Feng et al. (2021) also describe the role of small RNAs, which are major regulators of plant gene expression, for rice pathogens and insects. This Special Issue also showcases primary research and specific advances in molecular rice susceptibility and resistance research. Chung et al. (2020) use transcriptome analysis to define early defenses that might limit infection by the fungus *Fusarium fujikoroi*, which is the causal agent of bakanae disease and can cause losses of up to 50% for growers. Dong et al. determine a family of oxolate oxidases that positively regulate resistance, which may provide specific targets for genome editing against another fungal disease called panicle blast caused by *Magnaporthae oryzae*. Overall this comprehensive set of articles covers the spectrum of research advancing our molecular understanding of rice confronting biotic stress.

Plant resistance to pathogens depends on durable genetic traits and translating these discoveries to growers for sustainable management. This Special Issue showcases the dedication of multiple teams to define robust resistance against rice pathogens. Lu et al. (2021) build on the 3000 Rice Genomes Project to discover resistance loci against bacterial blight caused by *Xanthomonas oryzae* pv. *oryzae*, which is arguably the most impactful prokaryotic pathogen of rice. Liu and colleagues use genome-wide associations to determine the molecular mechanisms defining negative regulation of bacterial blight resistance. Finally, Huang et al. (Ni et al. 2021) work towards translating specific resistance with genetic engineering against both bacterial blight and leaf streak diseases. These articles provide promise for translating our knowledge to limit important diseases of rice.

2020 was the UN International Year of Plant Health, and this current Special Issue highlights work at the forefront of rice disease research. These inspiring advances will hopefully provide a base for where we can hope to continue research on the world’s most important food crop to feed the growing population.
